# Histone H4 aggravates inflammatory injury through TLR4 in chlorine gas-induced acute respiratory distress syndrome

**DOI:** 10.1186/s12995-020-00282-z

**Published:** 2020-10-08

**Authors:** Yanlin Zhang, Jian Zhao, Li Guan, Lijun Mao, Shuqiang Li, Jinyuan Zhao

**Affiliations:** 1 Research Center of Occupational Medicine, Peking University Third Hospital, No.49 North Garden Road, Haidian District, Beijing, 100191 China; 2 State Key Laboratory of Toxicology and Medical Countermeasures, Institute of Pharmacology and Toxicology, Academy of Military Medical Sciences, No.27 Taiping Road, Haidian District, Beijing, 100850 China

**Keywords:** Chlorine gas, Acute respiratory distress syndrome, Histone H4, Toll-like receptor, Pulmonary inflammation

## Abstract

**Background:**

Chlorine gas (Cl_2_) exposure remains a public health concern in household, occupational, and transportation accidents around the world. The death rate associated with acute respiratory distress syndrome (ARDS) caused by high concentrations of Cl_2_ is very high, mainly because the pathogenesis of ARDS remains unclear. Histone H4 has been identified as an important endogenous pro-inflammatory molecule. The present study aimed to examine the pathogenic role of histone H4 in Cl_2_-induced ARDS.

**Methods:**

ARDS was induced by Cl_2_ exposure in male C57BL/6 mice. Circulating histone H4, blood gas, pulmonary edema, endothelial activation, and neutrophil infiltration were measured during acute lung injury (ALI). Histone H4 or anti-H4 antibody was administered through the tail vein 1 h prior to Cl_2_ exposure to study the pathogenic role of histone H4. Toll-like receptor 2 knock-out (*Tlr2*-KO) and *Tlr4*-KO mice were used in conjunction with blocking antibody against TLR1, TLR2, TLR4, or TLR6 to explore the mechanism involved in histone H4-mediated injury.

**Results:**

Cl_2_ exposure induced a concentration-dependent ALI. The levels of circulating histone H4 were positively correlated with Cl_2_ concentrations. Pretreatment with intravenous histone H4 further aggravated lethality rate, blood gas, endothelial activation, and neutrophil infiltration, while anti-H4 antibody showed protective effects. *Tlr4* deficiency improved lethality rate, blood gas, and pulmonary edema, and prevented endothelial and neutrophil activation caused by Cl_2_ exposure. More importantly, *Tlr4* gene deletion greatly diminished the effect of histone H4 or anti-H4 antibody observed in wild-type (WT) mice. The impact of *Tlr2* on inflammatory injury was not significant. The role of TLRs was also validated by endothelial activation mediated by histone H4 in vitro.

**Conclusions:**

Circulating histone H4 played a pro-inflammatory role in ARDS caused by Cl_2_. TLR4 was closely involved in histone H4-mediated inflammatory injury. Therefore, intervention targeting histone H4 is potentially protective.

## Background

Chlorine gas (Cl_2_) exposure remains a public health concern in household, occupational, and transportation accidents around the world. It has also been used as a chemical warfare agent and weapon of mass destruction by terrorists [1, 2]. High concentrations of Cl_2_ can result in acute respiratory distress syndrome (ARDS), which manifests as pulmonary edema, hypoxemia, and uncontrolled overwhelming inflammation [3, 4].

The death rate associated with ARDS is very high, primarily because the interventions are mostly supportive and symptom-oriented. This is because the pathogenesis and underlying causes of ARDS remains unclear [5–7]. Respiratory system damage inflicted by Cl_2_ can be divided into two phases. The first phase is direct chemical toxicity to the airway, and the second is the more severe ensuing inflammatory response, which can result in ARDS. Few studies have assessed how direct injury triggers uncontrolled, overwhelming inflammation [8, 9].

Extracellular histones increase during acute inflammatory diseases such as trauma, aspiration, and sepsis, which can mediate distant organ damage [10, 11]. The cytotoxicity of extracellular histones is primarily due to histone H4 [12, 13].

This work aimed to study the pathogenic role of circulating histone H4 in Cl_2_-induced ARDS.

## Materials and methods

### Chemicals and reagents

Histone H4 was purchased from Millipore (Billerica, MA, USA). Blocking antibodies against TLR1 (GD2.F4), TLR2 (TL2.1), and TLR4 (HTA125) were purchased from eBioscience (San Diego, CA, USA). Antibodies for TNF-α, IL-1β, P-selectin, Ly6G, H4, Ac-H4(acetyl K5 + K8 + K12 + K16) and blocking antibody against TLR6 (TLR6.127) were purchased from Abcam (Cambridge, UK). An ELISA kit for von Willebrand factor (vWF) and a myeloperoxidase (MPO) detection kit were purchased from Jiancheng Biotech (Nanjing, China). The blocking antibody against histone H4 (anti-H4) was prepared following the previously described protocol involving autoimmune mice [14].

### Animals

Eight-week-old wild-type (WT) C57BL/6 mice, Toll-like receptor 2 knock-out (*Tlr2*-KO) mice and *Tlr4*-KO mice were provided by Peking University Animal Center (Beijing, China). The *Tlr4* gene (NCBI Reference Sequence: NM_021297) is located on mouse chromosome 4. Exon 2 was selected as the target site among the three exons identified. The *Tlr2* gene (NCBI Reference Sequence: NM_011905) is located on mouse chromosome 3. Exon 3 was selected as the target site among the three exons identified. Cas9 and gRNA were co-injected into fertilized eggs for KO mouse production. The genotype was confirmed by polymerase chain reaction (PCR) and sequencing analysis [15]. All the genetically deficient mice were backcrossed at least six generations onto the B6 background before the experiment. The protocols for the animal experiment were reviewed and permitted by the Peking University Animal Care and Use Committee (No. LA201783). Mice were housed in an air-conditioned room at 25 °C with a 12 h dark-light cycle. To minimize animal suffering, mice were administered anesthesia before surgery and euthanized humanely by intravenous injection of xylazine (6 mg/kg) and ketamine (90 mg/kg) followed by cervical dislocation.

### Chlorine gas exposure

Cl_2_ exposure was performed in a special chamber (no more than six mice at a time) where Cl_2_ released from a cylinder was mixed with air as previously described [16]. The concentration of Cl_2_ was monitored by a chlorine detector. When the Cl_2_ concentration decreased, the Cl_2_-air mixture was immediately replaced. The exposure conditions were 10, 20, 50, 100, 200, 400, 600, and 800 ppm respectively for 30 min. The control mice stayed in the same chamber for 30 min without Cl_2_. Immediately after Cl_2_ exposure, mice were returned to room air. They were monitored hourly for 12 h, and every 4 h thereafter for 72 h. The mouse model of Cl_2_-induced ARDS was validated by blood gas analysis (PaO_2_/FiO_2_ ≤ 300 mmHg).

### Measurement of circulating histone H4 and blood gas analysis

To obtain blood samples, a catheter with 11 mM sodium citrate was inserted into the abdominal aorta after the mice were anesthetized. To measure arterial partial oxygen pressure (PaO_2_), whole blood (0.1–0.2 ml) was measured with a blood gas analyzer (Ciba Corning, Canada). To measure histone H4, the plasma was collected from the whole blood after centrifugation and then analyzed with a histone H4 detection kit (USCN, Wuhan, China).

### Cell culture

Human pulmonary vascular endothelial cells were purchased from the Peking Union Medical College (Beijing, China). They were cultured in Dulbecco’s Modified Eagle Medium (DMEM) with 10% fetal bovine serum (FBS) at 37 °C. To determine which TLR was involved, a blocking antibody (10 mg/L) against TLR1, TLR2, TLR4, or TLR6 was concurrently administered when the endothelial cells were stimulated by histone H4 (10 mg/L) for 12 h. Each group was tested in triplicate at each time point.

### Real-time PCR

Quantitative PCR was performed with *TNFA* (MP200370) and *IL1B* (MP200131) primers (Sino Biological, Radnor, PA, USA). The reverse transcription reactions were conducted with a Cells-to-CT kit (Invitrogen, Carlsbad, CA, USA) in 7300 Real-Time PCR System (AB Biosciences, Concord, MA, USA). PCR conditions included initial denaturation at 95°Cfor 10 min, followed by 40 cycles of denaturation at 95 °C for 15 s, and annealing at 60 °C for 1 min. *GAPDH* was used as the housekeeping gene. The relative abundance of mRNA expression in each sample was calculated as 2^-∆∆Ct^.

### Western blot

Protein concentration was measured by the Bio-Rad Protein Assay Kit. For all groups, proteins (40 μg) were mixed with loading buffer. After electrophoresis, the separated proteins were transferred onto PVDF membranes. The membranes were probed with anti-TNF-α antibody (1:2000), anti-IL-1β antibody (1: 2000), anti-H4 antibody (1: 1000), and anti-GAPDH antibody (1: 5000) overnight at 4 °C.

### Statistical analysis

The data are shown as mean ± standard deviation (SD) and analyzed with Prism (GraphPad Software). Pearson correlation analysis was used to analyze correlation and a log-rank test was used to analyze survival rate. An analysis of variance (ANOVA) was used to analyze the statistical differences among groups and the Student-Newman-Keuls test was used to analyze the differences between groups. A *p*-value < 0.05 was viewed as statistically significant.

## Results

### Circulating histone H4 increased in Cl_2_-induced ARDS in mice

Different concentrations of Cl_2_ (10, 20, 50, 100, 200, 400, 600, 800 ppm) were used to induce ARDS in mice (*n* = 12, exposure time 30 min). The effect of Cl_2_ on the severity of ALI was concentration dependent. Low Cl_2_ concentrations (≤ 100 ppm) merely caused brief tachypnea, and the mice (12/12) all survived. When the concentration increased to 200 ppm, Cl_2_ caused dyspnea and eight mice (8/12) survived for 72 h. A concentration of 400 ppm caused evident dyspnea, and five mice (5/12) survived for 72 h. A concentration of 600 ppm caused serious dyspnea and only three mice (3/12) survived for 72 h. Almost, all mice (11/12) died within 72 h after exposure to a Cl_2_ concentration of 800 ppm. Pulmonary interstitial edema, inflammatory cell infiltration, hemorrhage, atelectasis, microthrombus formation, and epithelium necrosis were evident. The pathological changes in lung tissue 24 h after Cl_2_ exposure were shown in Figure S1 (supplemental data).

**Fig. 1:**
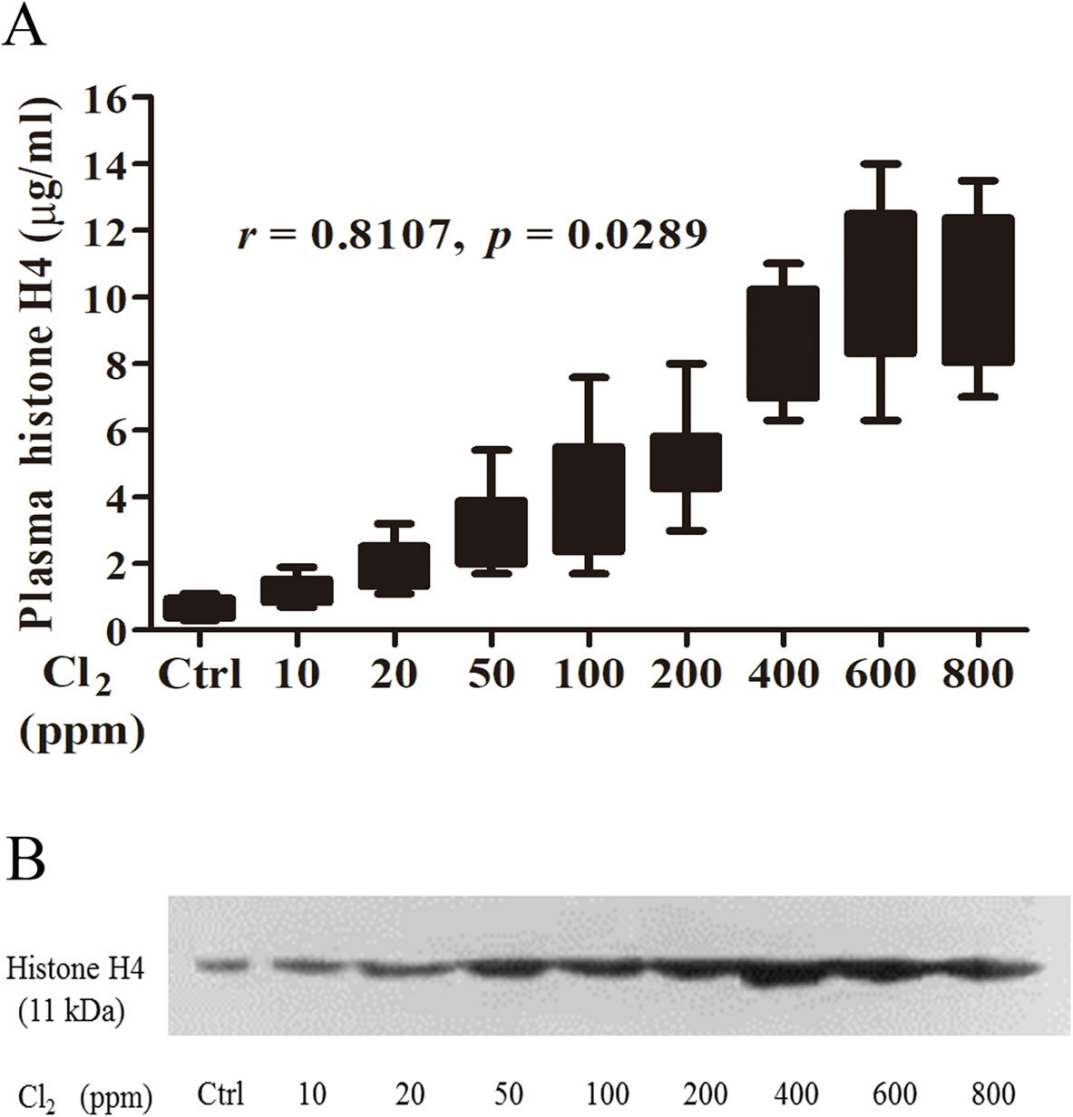
Change of circulating histone H4 in Cl_2_-induced ARDS. Twenty four hours after mice were treated (30 min) with different concentrations of Cl_2_, circulating histone H4 was measured with an ELISA kit (**a**) and western blot (**b**). Data were presented as mean ± SD (*n* = 6). Western blot was performed in triplicate

Circulating histone H4 was very low in the normal state. After Cl_2_ exposure, circulating histone H4 increased, particularly when the concentration of Cl_2_ reached 400 ppm. As shown in Fig. 1a, there was a significant positive correlation between the concentrations of Cl_2_ (from 10 to 800 ppm) and histone H4 in plasma (*r* = 0.8017, *p* = 0.0289). As shown in Fig. 1b, western blot was used to further prove the histone H4 changes in the plasma.

### Effects of histone H4 and TLRs on survival rate

**Fig. 2:**
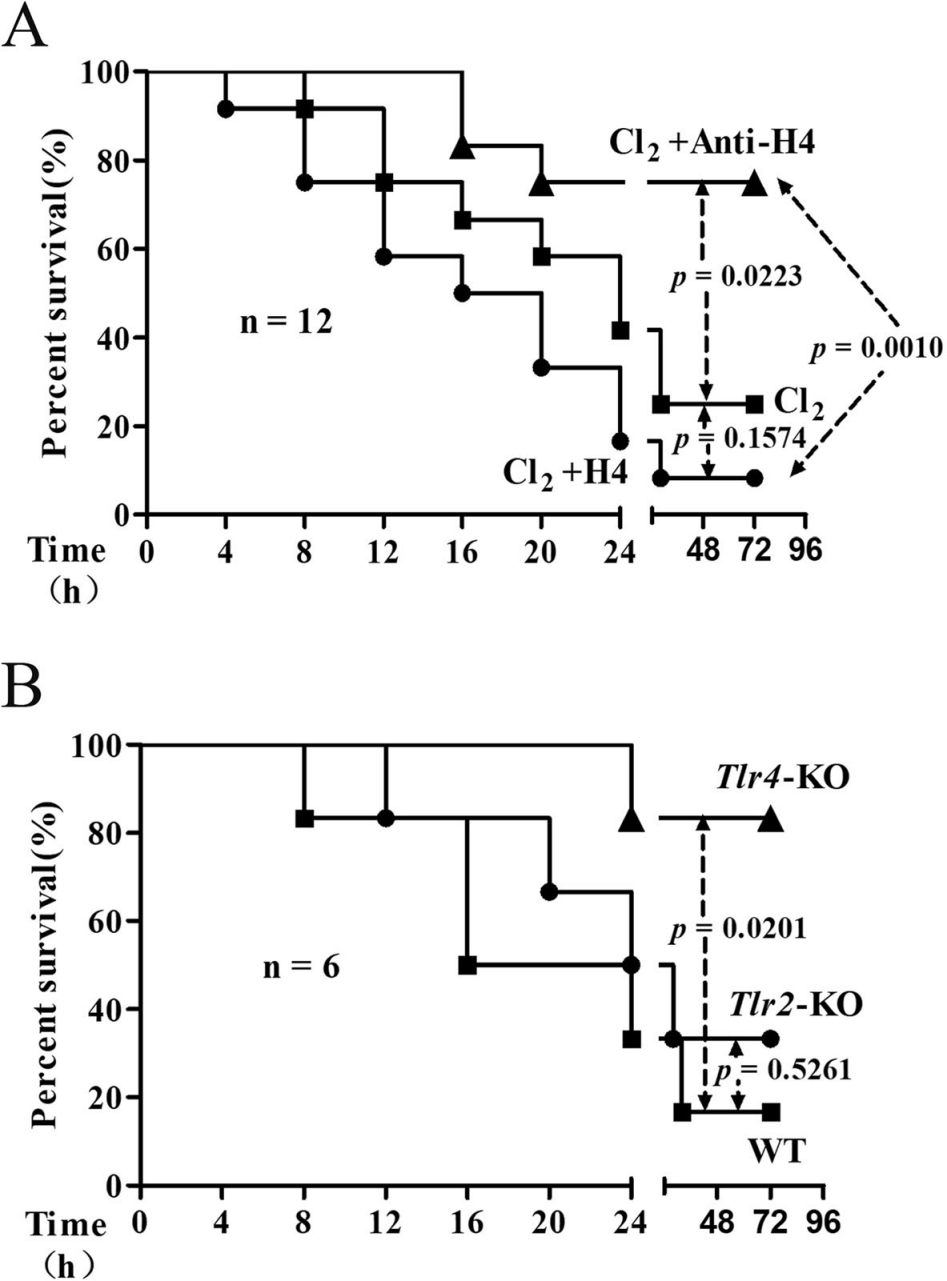
Effects of histone H4 and TLRs on survival rate. After exposure to lethal concentration of Cl_2_ (600 ppm, 30 min), the survival rate was recorded at predetermined time points (every 4 h for 96 h). Histone H4 (10 mg/kg) or anti-H4 antibody (20 mg/kg) was delivered through the tail vein 1 h prior to Cl_2_ exposure in the WT mice (*n* = 12, 2A). *Tlr2*-KO and *Tlr4*-KO mice were used to explore the role of TLRs (n = 6, 2B). The *p* values are compared with the WT mice treated solely with Cl_2_

In order to study the role of histone H4, mice were injected via tail vein with histone H4 or anti-H4 antibody 30 min prior to Cl_2_ exposure. As shown in Fig. 2a, nine mice (9/12) died within 72 h after exposure to lethal Cl_2_ concentration (600 ppm, 30 min). When pretreated with intravenous histone H4 (10 mg/kg), nearly all mice (11/12) died within 72 h after Cl_2_ exposure (*p* = 0.1574, compared with the mice solely exposed to Cl_2_). Only three mice (3/12) died when pretreated with intravenous anti-H4 antibody (20 mg/kg) (*p* = 0.0223, compared with the mice solely exposed to Cl_2_). The difference in lethality rate between the mice pretreated with histone H4 and those pretreated with anti-H4 antibody was statistically significant (*p* = 0.0010).

*Tlr2*-KO and *Tlr4*-KO mice were used to investigate the role TLRs in ARDS. As shown in Fig. 2b, five WT mice (5/6) died within 72 h after exposure to lethal Cl_2_ concentration (600 ppm, 30 min). Compared with the WT mice, four *Tlr2*-KO mice (4/6) and only one *Tlr4*-KO mouse (1/6) died within 72 h after Cl_2_ exposure. The difference in lethality rate between the WT mice and the *Tlr4*-KO mice was statistically significant (*p* = 0.0201). Thus, the *Tlr2*-KO and *Tlr4*-KO mice were resistant to Cl_2_ exposure of lethal concentrations to some extent.

### Roles of histone H4 and TLRs in Cl_2_-induced ALI

**Fig. 3:**
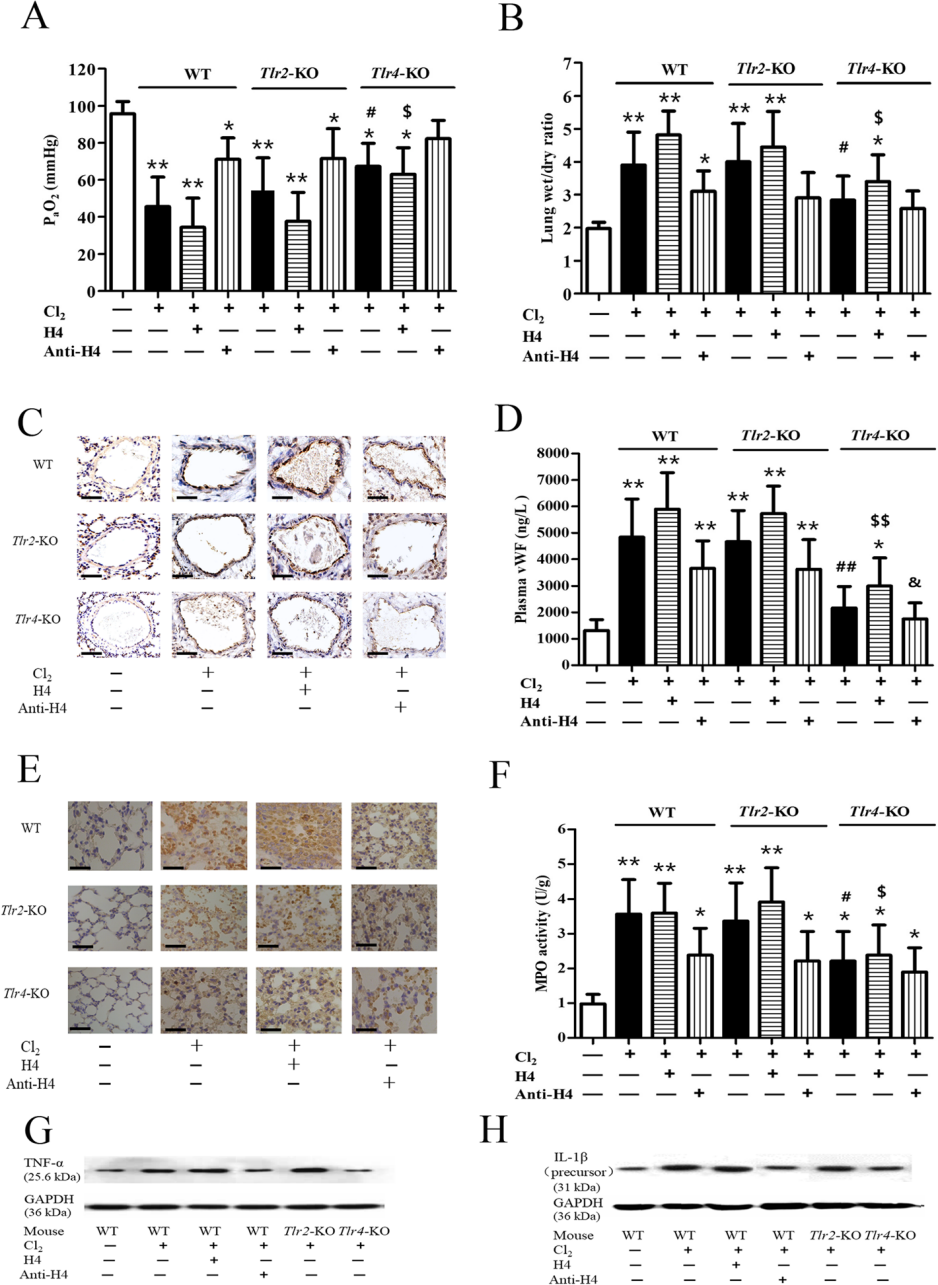
Roles of histone H4 and TLRs in Cl_2_ induced inflammatory lung injury. Twenty four hours after Cl_2_ exposure (400 ppm, 30 min) in WT, *Tlr2*-KO, and *Tlr4*-KO mice, PaO_2_ (3A) and lung wet/dry mass ratio (3B) were measured. Pulmonary endothelial activation was represented by P-selectin expression (3C) (*Scale bars*: 100 μm) and circulating vWF (3D). Pulmonary neutrophil infiltration was shown by Ly6G marker staining (3E) (*Scale bars*: 50 μm), and neutrophilic activation was indicated by MPO activity (3F). The protein levels of inflammatory cytokines TNF-α (3G) and IL-1β (3H) were measured by western blot. Histone H4 (10 mg/kg) or anti-H4 antibody (20 mg/kg) was delivered through the tail vein 1 h prior to Cl_2_ exposure. *N* = 6 independent replicates for all groups. *p < 0.05, **p < 0.01 compared with the control group; ^#^*p* < 0.05, ^##^*p* < 0.01 compared with the WT mice treated solely with Cl_2_; ^$^*p* < 0.05, ^$$^*p* < 0.01 compared with the WT mice treated in the same manner (Cl_2_ + H4); ^&^*p* < 0.05, ^&&^*p* < 0.01 compared with the WT mice treated in the same manner (Cl_2_ + Anti-H4).

As shown in Fig. 3a, Cl_2_ exposure caused obvious hypoxemia (48.73 ± 14.23 mmHg) in the WT mice (*p* = 0.0083, compared with the control group). In mice pretreated with intravenous histone H4 (10 mg/kg), PaO_2_ decreased much more seriously to 36.35 ± 15.17 mmHg (*p* = 0.0067, compared with the control group). On the contrary, PaO_2_ increased to 70.35 ± 11.51 mmHg (*p* = 0.0474, compared with the control group) when mice were pretreated with intravenous anti-H4 antibody (20 mg/kg). Compared with the WT mice treated in the same manner, PaO_2_ in the *Tlr4*-KO mice only decreased slightly to 67.31 ± 10.66 mmHg 24 h after Cl_2_ exposure (*p* = 0.0443). More importantly, the damaging effect of histone H4 was diminished greatly in the *Tlr4*-KO mice compared with the WT mice treated in the same manner (*p* = 0.0268). Furthermore, the effect of the anti-H4 antibody was also decreased in the *Tlr4*-KO mice. In contrast to the *Tlr4*-KO mice, the PaO_2_ changes and the effects of histone H4 or anti-H4 antibody in the *Tlr2*-KO mice were similar to those in the WT mice.

As shown in Fig. 3b, pulmonary edema was serious after Cl_2_ exposure in the WT mice (*p* = 0.0092, compared with the control group). Pretreatment with histone H4 (10 mg/kg) further increased the lung wet/dry mass ratio to 4.81 ± 0.97 (*p* = 0.0073, compared with the control group). However, pretreatment with anti-H4 antibody (20 mg/kg) decreased the lung wet/dry mass ratio to 3.16 ± 0.68 (*p* = 0.0319, compared with the control group). Compared with the WT mice treated in the same manner, the lung wet/dry mass ratio in the *Tlr4*-KO mice merely increased to 2.96 ± 0.71 (*p* = 0.0457) after Cl_2_ exposure. Additionally, the damaging effect of histone H4 was diminished in the *Tlr4*-KO mice compared with the WT mice treated in the same manner (*p* = 0.0338). The effect of anti-H4 antibody was also decreased. In contrast to the *Tlr4*-KO mice, the *Tlr2*-KO mice did not show obvious differences in pulmonary edema when compared with the WT mice.

Cl_2_ exposure caused evident endothelial activation that manifested as elevated P-selectin expression (Fig. 3c) and vWF release from endothelial Weibel-Palade bodies (WPBs) (Fig. 3d) (*p* = 0.0038, compared with the control group). Pretreatment with intravenous histone H4 (10 mg/kg) aggravated P-selectin expression and caused further release of vWF (*p* = 0.0017, compared with the control group). However, pretreatment with the anti-H4 antibody (20 mg/kg) produced some protective effects on P-selectin overexpression and vWF release (*p* = 0.0089, compared with the control group). Compared with the WT mice treated in the same manner, P-selectin overexpression and vWF release in the *Tlr4*-KO mice were decreased (*p* = 0.0093) after Cl_2_ exposure. The effects of histone H4 (*p* = 0.0075) and anti-H4 antibody (*p* = 0.0377) were diminished greatly in the *Tlr4*-KO mice compared with the WT mice treated in the same manner. However, changes in endothelial activation and the effect of histone H4 or anti-H4 antibody in the *Tlr2*-KO mice were similar to those in the WT mice.

Pulmonary neutrophil infiltration and activation were prominent after Cl_2_ exposure, and were measured by using neutrophil specific marker Ly6G (Fig. 3e) and MPO activity (Fig. 3f) (*p* = 0.0031, compared with the control group). Pretreatment with intravenous histone H4 (10 mg/kg) increased neutrophil infiltration and MPO activity further (*p* = 0.0017, compared with the control group) while pretreatment with anti-H4 antibody (20 mg/kg) showed some antagonistic effects (*p* = 0.0165, compared with the control group). Compared with the WT mice treated in the same manner, neutrophil infiltration and activation in the *Tlr4*-KO mice were inhibited after Cl_2_ exposure (*p* = 0.0234). The effects of histone H4 (*p* = 0.0341) and anti-H4 antibody (*p* = 0.0972) were also diminished in the *Tlr4*-KO mice compared with the WT mice treated in the same manner. In contrast to the *Tlr4*-KO mice, the *Tlr2*-KO mice did not show evident differences in neutrophil infiltration and activation compared with the WT mice.

As shown in Fig. 3g and h, Cl_2_ exposure triggered the expression of inflammatory cytokines, such as TNF-α and IL-1β, in WT mice compared with the control group. Pretreatment with intravenous histone H4 (10 mg/kg) further increased the protein level of TNF-α and IL-1β. However, pretreatment with anti-H4 antibody (20 mg/kg) inhibited the protein expression of TNF-α and IL-1β caused by Cl_2_ exposure. In the *Tlr4*-KO mice, the protein level of TNF-α and IL-1β was much lower than that in the WT mice. In contrast to the *Tlr4*-KO mice, the *Tlr2*-KO mice did not show obvious differences in TNF-α and IL-1β protein expression compared with the WT mice.

### TLRs involved in histone H4-mediated endothelial inflammation in vitro

**Fig. 4:**
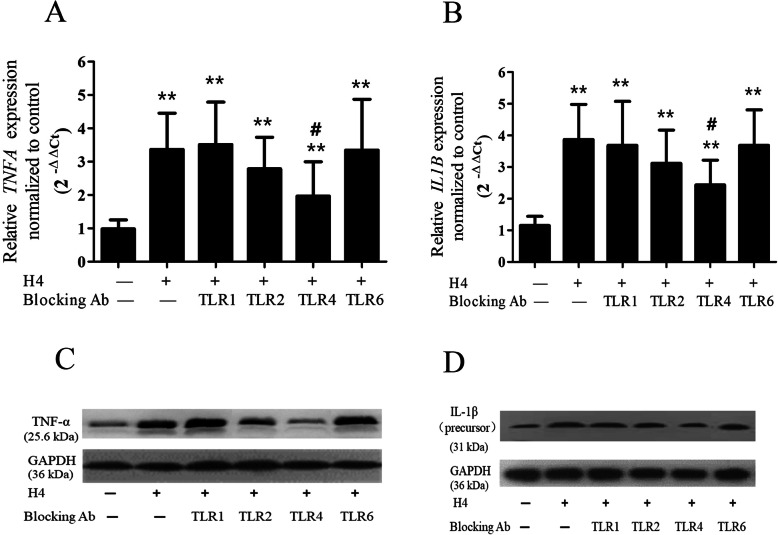
TLRs involved in histone H4 mediated expression of inflammatory cytokines. The blocking antibody (10 mg/L) against TLR1, TLR2, TLR4, or TLR6 was concurrently administered when pulmonary vascular endothelial cells were challenged by histone H4 (10 mg/L) for 12 h. The mRNA of *TNFA* and *IL1B* was measured by real-time qPCR (**a**, **b**), and protein was measured by western blot (**c**, **d**). *N* = 3 independent replicates for all groups. *p < 0.05, **p < 0.01 compared with the control group; ^#^*p* < 0.05, ^##^*p* < 0.01 compared with the histone H4 group

Blocking antibodies against TLR1, TLR2, TLR4, and TLR6 were used to investigate the role of TLRs in histone H4-mediated endothelial inflammation. Histone H4 (10 mg/L) treatment triggered the expression of inflammatory cytokines in the pulmonary vascular endothelial cells, including TNF-α and IL-1β. As shown in Fig. 4a, a blocking antibody against TLR4 distinctly reduced the transcription of *TNFA* (38% decrease versus H4 group, *p* = 0.0373). Additionally, blocking antibody against TLR2 slightly reduced the transcription of *TNFA* (*p* > 0.05). However, blocking antibodies against TLR1 and TLR6 showed little effect. The effects of the blocking antibodies against TLR1, TLR2, TLR4, and TLR6 on TNF-α protein expression were similar to their effects on transcription (Fig. 4c).

As shown in Fig. 4b, the blocking antibody against TLR4 markedly reduced the transcription of *IL1B* (41% decrease versus H4 group, *p* = 0.0211), while the effect of the blocking antibody against TLR2 on *IL1B* transcription was much weaker (*p* > 0.05). The blocking antibodies against TLR1 and TLR6 showed little effect on *IL1B* transcription. The effect of the blocking antibodies on IL-1β was also validated by protein expression (Fig. 4d).

## Discussion

Exposure to high levels of Cl_2_ remains a documented public threat. The severity of Cl_2_-induced injury depends primarily on the concentration and duration of exposure [3]. ARDS is the most severe consequence, and the overwhelming inflammatory response triggered by the initial injury is considered as the ARDS keystone [17, 18].

In addition to the classic pathogen-associated molecular patterns (PAMPs), damage-associated molecular patterns (DAMPs) are another significant pathway of uncontrolled inflammation. Extracellular histones were recognized as DAMP molecules in 2009 [12], and are essential inflammatory mediators in a variety of diseases such as sepsis, trauma, and multiple organ dysfunction syndrome [19, 20].

In this study, it was shown that Cl_2_ induced concentration-dependent lung injury. The levels of circulating histone H4 were closely correlated with Cl_2_ concentrations (ranging from 10 to 800 ppm). The concentration of Cl_2_ was a major determinant for the level of circulating histone H4. The pathogenic role of histone H4 in Cl_2_-induced ARDS was proven by histone H4 or specific blocking anti-H4 antibody intervention. Pretreatment with histone H4 further worsened the lethality rate and PaO_2_, while anti-H4 antibody showed some protective effects. Compared with the control mice, both endothelial and neutrophil activation were much more distinct in mice with Cl_2_-induced ARDS, which was indicated by P-selectin expression, vWF release, pulmonary neutrophil infiltration, and elevated MPO activity. Pretreatment with intravenous histone H4 aggravated endothelial and neutrophil activation, while anti-H4 antibody played an antagonistic role. Thus, it can be deduced that circulating histone H4 was a critical inflammatory mediator in Cl_2_-induced ARDS.

Additionally, TLRs were closely involved with histone H4-mediated inflammatory injury during Cl_2_-induced ARDS. The *Tlr4*-KO mice were resistant to exposure to lethal Cl_2_ concentrations. *Tlr4* deficiency improved lethality rate, PaO_2_, and pulmonary edema, and prevented the endothelial and neutrophil activation caused by Cl_2_ exposure. More importantly, *Tlr4* gene deletion greatly diminished the effect of histone H4 or anti-H4 antibody observed in WT mice. In contrast to *Tlr4* deficiency, the impact of *Tlr2* on inflammatory injury was not evident. In addition to TLR4 and TLR2, the roles of TLR1 and TLR6 were also screened. The blocking antibody against TLR4 or TLR2 decreased the histone H4-mediated expression of *TNFA* and *IL1B* in human pulmonary vascular endothelial cells, while the blocking antibody against TLR1 or TLR6 showed little effect.

Histones are structural elements of nuclear chromatin that mainly regulate gene expression. When histones are released passively from necrotic cells or actively by cell death such as NETosis, extracellular histones produce toxic effects on adjacent and circulating cells [21, 22]. The predominant source of histones may be neutrophils that have been activated by C5a to form neutrophil extracellular traps (NETs) in ALI [23, 24]. In contrast, nucleosomes, composed of histones and DNA, appear to be less toxic [25, 26]. Extracellular histones are DAMP molecules that can cause systemic inflammation involved in a wide range of inflammatory conditions, such as acute lung injury, liver injury, kidney injury, myocardial injury, cerebral stroke, coagulopathy, systemic lupus erythematosus, and even hair follicle death [27–34]. Extracellular histones can induce the release of chemokines, and activate the vascular endothelium, so as to facilitate leukocyte adhesion and transmigration [35, 36]. Both endothelial and neutrophilic activation are key events during the ARDS inflammatory response [37, 38]. Elevated P-selectin expression is an important marker for endothelial activation. Additionally, P-selectin translocation is a prerequisite for neutrophil adhesion to the pulmonary vascular endothelium during inflammation [39, 40]. Along with P-selectin translocation, abundant vWF is released from the endothelia, which excessively activates the coagulation cascade and aggravates lung injury [41, 42]. In addition to the endothelia and neutrophils, Westman et al. showed that extracellular histones induced monocytes to produce chemokines such as CXCL9 and CXCL10, which triggered neutrophil recruitment [36]. Fuchs et al. showed that extracellular histones bound to platelets, inducing calcium influx, recruiting plasma adhesion proteins such as fibrinogen, and triggering microaggregation [43].

Extracellular histones serve as DAMP molecules to promote inflammation. ARDS is an overwhelming inflammatory response that is triggered by various damaging factors. The initial detrimental stimuli are sensed by pattern recognition receptors (PRRs) [44]. TLRs are responsible for sensing invading pathogens and injury stimuli outside of the cell, as well as in intracellular endosomes and lysosomes. TLR1, TLR2, TLR4, TLR5, and TLR6 are present in the plasma membrane, while TLR3, TLR7, and TLR9 are mainly present in the membrane of the endoplasmic reticulum [45]. Thus, TLR1, TLR2, TLR4, TLR5, and TLR6 may mediate the inflammation caused by extracellular histones. In accordance with the results of this study, both TLR2 and TLR4 were previously found to be involved with extracellular histones-induced liver and kidney injury [39, 46]. In addition to TLR2 and TLR4, other PRRs are also involved in extracellular histones-induced inflammation. Huang et al. demonstrated that *Tlr9*-KO mice were protected from histone-mediated ischemia/reperfusion-induced liver injury [47]. TLR9 is generally viewed as a receptor mediating signaling brought about by endogenous circulating DNA released from dying cells. The exogenous histones may act as a cofactor of DNA, and thus, they can amplify TLR9-mediated signaling.

Histones are unique cytotoxic DAMP molecules that elicit both PRR-dependent pro-inflammatory signaling and PRR-independent direct cytotoxicity. Rationally, the affinity of histones for phosphodiester bonds may ensure their avid binding to both DNA in the nucleus and phospholipids in the plasma membrane [48, 49]. Abrams et al. demonstrated that FITC-labeled histones directly bind to the surface of cultured EA. hy926 endothelial cells, subsequently inducing an influx of calcium, ultimately resulting in cell lysis [11]. Silvestre-Roig et al. demonstrated that when smooth muscle cells (SMCs) were exposed to histone H4, alterations in the membrane were characterized by dynamic bending and pore formation. The activated SMCs attracted neutrophils and triggered the ejection of NETs. NETs, or histone H4 could induce SMC swelling and release of ATP. Finally, extracellular histone H4 triggered inflammation and arterial tissue damage by mediating SMC membrane lysis [50].

It is well-known that histone-acetylation (open chromatin) promotes gene expression, whereas histone deacetylation (closed chromatin) represses gene expression in living cells [51, 52]. To clarify whether extracellular histones underwent acetylation modification, this study detected histone H4 in plasma samples by western blot using an anti-acetylated histone H4-antibody (K5, K8, K12, K16). As shown in Figure S2, the acetylation status of plasma histone H4 in Cl_2_ exposure groups (10, 50 ppm) was similar to its status in the control group. An increase in the acetylation level of plasma histone H4 in the Cl_2_ exposure groups (200, 800 ppm) was not evident compared with the control group. Extracellular histones are released by NETs and apoptotic/necrotic cells. The main role of histones released from NETs is to kill bacteria instead of regulating gene transcription. Thus, acetylation modification was not applicable to them [53]. For the histones released from apoptotic/necrotic cells, they might be acetylated before release and retained the acetylation modification after release. It can be deduced that the pro-inflammatory effect of extracellular histones is mainly concentration dependent.

Because extracellular histones are cytotoxic DAMP molecules, it is rational that the damaging effects can be ameliorated through the application of histone-targeted interventions [54]. Administration of specific blocking antibodies or peptides targeted to these extracellular histones could inhibit inflammatory damage and improve outcomes in several types of animal models, such as sepsis, acute lung injury, liver injury, acute pancreatitis, and multiple organ injury [10, 55–58]. The direct toxicity of extracellular histones is dependent on the electrostatic membrane’s interaction with target cells. Accordingly, histone neutralizing agents have been identified as therapeutic options in treating extracellular histone-mediated tissue injury. Heparin is a highly sulfated negatively charged proteoglycan. Heparin and its derivatives can bind histones through electrostatic interactions, and they have demonstrated the ability to inhibit ALI, vascular endothelial injury, and thrombocytopenia caused by histones released [59, 60]. In addition to heparin and its derivatives, many innate and synthetic substances have been proven to prevent histone-related toxicity, including plasma albumin, C-reactive protein, bacterial O-antigen, polyglutamic acid and polysialic acid [25, 61–64].

## Conclusions

This study showed that circulating histone H4 played a pro-inflammatory role primarily through TLR4 in ARDS caused by Cl_2_. This may help to explain the pathogenesis of ARDS and promote potentially effective intervention. Surely, the mechanisms for Cl_2_-induced inflammatory injury are very complex, so more thorough investigation is required to further understand the pathogenesis. Since the data originate from animal experiments, caution must be exercised in extrapolating these findings to more complicated clinical situations.

## Supplementary information

**Additional file 1: Figure S1.** Pathological changes in lung tissue after Cl_2_ exposure. Twenty four hours after exposure (30 min) to different concentrations (10 to 800 ppm) of Cl_2_, pulmonary histopathology was analyzed, including alveolar hemorrhage, interstitial edema, infiltration of inflammatory cells, and disruption of the alveolar wall. Lung sections were stained with hematoxylin and eosin (HE). *N* = 6 for all groups. *Scale bars*: 100 μm. **Figure S2** Acetylation analysis of circulating histone H4 after Cl_2_ exposure. Twenty four hours after mice were treated (30 min) with different concentrations (10, 50, 200, and 800 ppm) of Cl_2_, the acetylation status of circulating histone H4 was analyzed by western blot. The concentration of histone H4 was measured with a histone H4 detection kit. Equal amounts of histone H4 were mixed with loading buffer and subjected to electrophoresis. Western blot was performed in triplicate.

## Data Availability

The datasets used and/or analyzed during the current study are available from the corresponding author on reasonable request.

## Abbreviations

ALI: Acute lung injury
ARDS: Acute respiratory distress syndrome
Cl_2_: Chlorine gas
PaO_2_: Arterial partial oxygen pressure
MPO: Myeloperoxidase
vWF: von Willebrand factor
WPBs: WEIBEL-Palade bodies
PAMPs: Pathogen-associated molecular patterns
DAMPs: Damage-associated molecular patterns
PRRs: Pattern recognition receptors
TLR: TOLL-like receptor
WT: Wild-type
KO: Knock-out
