# Supplementary figures and images for: Histone H4 aggravates inflammatory injury through TLR4 in chlorine gas-induced acute respiratory distress syndrome
Source: J Occup Med Toxicol. 2020 Oct 8;15:31. doi: 10.1186/s12995-020-00282-z (PMC7545935; doi:10.1186/s12995-020-00282-z)

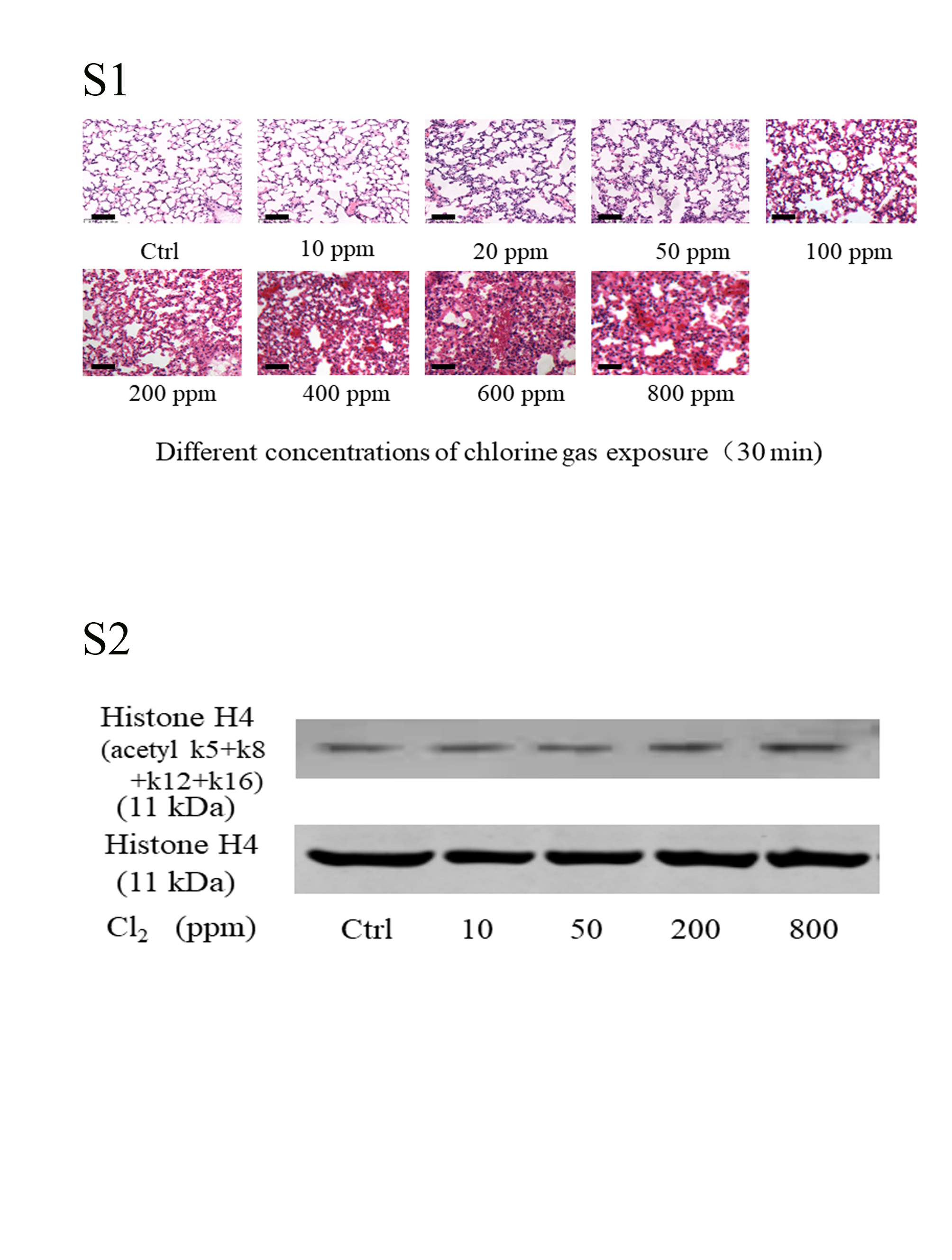

Supplement: Supplementary file 1 — Additional file 1: Figure S1. Pathological changes in lung tissue after Cl2 exposure. Twenty four hours after exposure (30 min) to different concentrations (10 to 800 ppm) of Cl2, pulmonary histopathology was analyzed, including alveolar hemorrhage, interstitial edema, infiltration of inflammatory cells, and disruption of the alveolar wall. Lung sections were stained with hematoxylin and eosin (HE). N = 6 for all groups. Scale bars: 100 μm. Figure S2 Acetylation analysis of circulating histone H4 after Cl2 exposure. Twenty four hours after mice were treated (30 min) with different concentrations (10, 50, 200, and 800 ppm) of Cl2, the acetylation status of circulating histone H4 was analyzed by western blot. The concentration of histone H4 was measured with a histone H4 detection kit. Equal amounts of histone H4 were mixed with loading buffer and subjected to electrophoresis. Western blot was performed in triplicate. [file 12995_2020_282_MOESM1_ESM.tif]
